# Preparation and long-term corrosion resistance of epoxy resin coatings modified by Fe-Co-Ni-Cr-Ti high-entropy alloy

**DOI:** 10.1039/d6ra00478d

**Published:** 2026-03-27

**Authors:** Xinyue Fan, Yaqian Liu, Guojun Ji

**Affiliations:** a College of Chemical Engineering, Inner Mongolia University of Technology Hohhot 010051 China jgj@imut.edu.cn lyq@imut.edu.cn; b Inner Mongolia Key Laboratory of Green Chemical Engineering Hohhot 01005l China; c Research Center for Special Equipment Protection and Inspection Engineering, Inner Mongolia University of Technology Hohhot 010051 China

## Abstract

Traditional epoxy resin coatings easily form pores and microcracks in corrosive media such as NaCl, resulting in insufficient long-term corrosion resistance. To solve this problem, this work fabricated low-cost Fe-Co-Ni-Cr-Ti high-entropy alloy (HEA)-modified novolac epoxy coatings (HEA/EPN) *via* optimized dispersion and preparation processes. HEA/EPN coatings with 1–4 wt% HEA were prepared. The effects of HEA content and dispersion on coating micromorphology and phase composition were characterized, and their long-term corrosion resistance was evaluated using electrochemical impedance spectroscopy (EIS). Results showed that nano-sized HEA not only filled pores to enhance the coating barrier effect but also induced *in situ* passive film formation to inhibit corrosion. HEA dispersion directly determined coating corrosion resistance, with the 2 wt% HEA coating exhibiting optimal performance—its charge transfer resistance (*R*_ct_) remained 4.27 × 10^8^ Ω cm^−2^ after 60 days immersion. This study provides a viable low-cost solution to the poor long-term corrosion resistance of conventional epoxy coatings. The as-prepared HEA-modified epoxy coating presents promising application prospects in marine engineering facilities, petrochemical pipelines and storage tanks, as well as metal components in infrastructure and transportation equipment under severe corrosive environments.

## Abstract 2

Fe-Co-Ni-Cr-Ti high-entropy alloy (HEA) modified epoxy coatings were prepared. The optimized 2 wt% HEA coating showed excellent long-term corrosion resistance in NaCl solution *via* a synergistic barrier-passivation mechanism.

## Graphical abstract

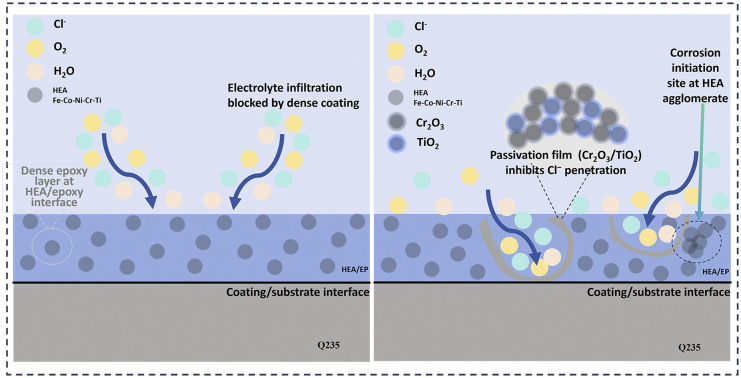

## 1 Introduction

Metallic materials are widely used in various industries due to their excellent inherent properties. However, their intrinsic susceptibility to corrosion renders them prone to destructive structural failure in harsh corrosive environments, which may lead to severe safety hazards and economic losses.^1–5^ Developing high-performance, cost-effective and durable metal anticorrosion protection technologies is critically important to both scientific research and engineering practice, as it can effectively ensure the safety of industrial operation and cut down economic losses substantially. Currently, commonly adopted metal anti-corrosion systems mainly include physical barrier coatings, electrochemical cathodic protection, elemental alloying, corrosion inhibitor addition, insulation protection, and corrosive environment improvement *via* inert gas purging.^6–8^

Among these strategies, epoxy resin coatings, as a typical type of physically barrier organic protective coatings, have long dominated the field of anti-corrosion coatings due to the abundant epoxy and hydroxyl groups on their molecular chains as well as their unique three-dimensional cross-linked network structure, which forms a dense barrier film after curing.^9–12^ However, with prolonged service time, epoxy resin coatings are subjected to the combined effects of external environmental erosion and intrinsic structural aging, which readily induce structural defects such as micropores and cracks inside the coatings. These defects provide diffusion pathways for the infiltration of corrosive media (*e.g.*, Cl^−^, O_2_, H^+^, H_2_O), triggering localized corrosion at the coating/metal interface and eventually resulting in coating degradation.^13–16^

To address this issue, researchers have recently attempted to incorporate multifunctional nanoparticles into epoxy resin matrices. Li *et al.*^17^ fabricated magnesium-aluminum layered double hydroxide (MgAl-LDH) nanosheets loaded with sodium phosphate (SP) corrosion inhibitors immobilized on their surface, which were then encapsulated in epoxy resin to fabricate an EP/Mg-Al@SP composite coating system. Electrochemical measurements indicated that the composite coating exhibited an impedance modulus three orders of magnitude greater than the pure epoxy (EP) coating. Xing *et al.*^18^ investigated the degradation process of epoxy resin coatings modified with zinc powder as anodic filler in 3.5% NaCl solution. Based on the variations in EIS spectra, the evolution of the coatings from intactness to failure was analyzed in detail. It was found that the barrier performance of the coatings decreased drastically after the complete consumption of zinc powder as the anodic component. Li *et al.*^19^ modified Ce^3+^ intercalated montmorillonite with tannic acid and dispersed the resulting Ce-MMT filler into waterborne epoxy resin to fabricate a multifunctional, high-efficiency anti-corrosion coating. The results showed that the low-frequency impedance modulus of the composite coating remained two orders of magnitude higher than that of the pure epoxy resin coating even after 50 days of immersion, where montmorillonite served dual functions as a physical barrier and nanocontainer. It is apparent that nanofiller introduction for building physical barrier layers can efficiently boost the barrier ability and corrosion resistance of coatings, yet their long-term corrosion resistance remains unsatisfactory.^20^

As a class of emerging materials, high-entropy alloys (HEAs) have garnered extensive attention since their simultaneous and independent development by Professor J. W. Yeh and Professor B. Cantor in 2004, owing to their exceptional corrosion resistance, wear resistance, high hardness, and high strength.^21–24^ Cantor *et al.* systematically investigated the microstructural evolution in equiatomic multicomponent alloys, laying a vital foundation for subsequent HEA research.^22^

In recent years, the application of high-entropy alloys (HEAs) as functional coatings or fillers has become a rapidly growing hotspot for advanced corrosion and wear protection, owing to their unique multi-element synergistic effects and excellent stability in extreme environments.^25,27^ For instance, Meng *et al.*^25^ prepared AlNbTiV-based HEA coatings on zirconium alloys *via* laser cladding, achieving exceptional corrosion and tribocorrosion resistance. Murakami *et al.*^26^ reported that electroplated CrCoNi medium-entropy alloy coatings exhibited superior wear and corrosion resistance, providing a promising substitute for conventional hard chromium coatings. Meng *et al.*^27^ further revealed that a stable passive film significantly improved the tribo-corrosion performance of Al_*x*_NbTiV-based HEA coatings, while Zhang *et al.*^28^ demonstrated that indium addition optimized the microstructure and enhanced the wear resistance of CrMoNiTiV HEA coatings.

These studies confirm the great potential of HEAs for high-performance corrosion-resistant coatings. However, most reports focus on HEA coatings prepared by laser cladding or electroplating. Systematic studies on the long-term corrosion mechanism, passive film evolution with HEA content, and low-cost preparation of HEA fillers for epoxy composite coatings remain insufficient.

Incorporating HEAs as fillers into epoxy resin coatings represents one of the effective approaches to address the deficiency in long-term corrosion resistance of coatings. Lin *et al.*^29^ designed dual-phase (AlCoCrFeNi)_100−*x*_Fe_*x*_ HEAs and found that their excellent performance was mainly attributed to the FCC-BCC structure. Iron can also form stable Fe–Cr solid solutions with chromium, improving the compatibility between the coating and substrate. S. Aravind *et al.*^30^ fabricated CoCrFeCuTi HEAs *via* ball milling and achieved excellent corrosion resistance on 304 stainless steel after friction stir processing and annealing.

Among the alloying elements, chromium acts as the important component for improving the corrosion resistance of coatings, since Cr^3+^ oxides and hydroxides contribute to the formation of a compact passive film. Different from traditional stainless steels that mainly rely on Cr element to form a passive film, the synergistic effect of multiple principal elements in HEAs further optimizes the compactness and stability of the passive film. Such a multi-element synergistic enhancement mechanism is exactly the distinctive “cocktail effect” of HEAs.^31^ By introducing Cr, Ti, Mo, and Nb, HEAs can form dense composite passive films that effectively inhibit localized corrosion.^32–34^ Nevertheless, the high cost and complex, stringent preparation processes of HEAs remain critical challenges. Therefore, it is of great significance to design and fabricate long-term corrosion-resistant coatings that not only possess the ability to form dense passive films and exhibit high corrosion resistance but also feature low cost and facile preparation procedures.

In this work, we innovatively designed a high-entropy alloy modified epoxy resin anticorrosive coating. This method features a simple preparation process, greatly reduced production cost, and excellent long-term protective performance. Through high-energy ball milling, mechanical blending, and electrostatic spraying, nano-sized Fe-Co-Ni-Cr-Ti high-entropy alloy with different contents was doped into phenolic epoxy resin to obtain the HEA-EPN nanocomposite coating. The chemical structure and morphology of the high-entropy alloy were analyzed by XRD, SEM, and TEM. The surface of the coatings with different filler contents was characterized by SEM-EDS, and the long-term corrosion resistance of the prepared coatings was evaluated by electrochemical impedance spectroscopy (EIS).

The high-entropy alloy modified epoxy resin coating developed in this study can be applied to the long-term corrosion protection of offshore platform steel structures in marine engineering. Offshore platforms are exposed to harsh marine environments for a long time, where chloride ions can easily cause local corrosion of steel structures. This coating can form a dense and stable passive film on the surface, which effectively blocks the penetration of corrosive media and provides reliable long-term corrosion protection for offshore platform components. In addition, this coating can also be extended to the inner wall protection of petrochemical equipment such as oil and gas pipelines and storage tanks.

This study innovatively provides an important theoretical basis and unique process technology support for the development and application of high-entropy alloys as fillers for modified epoxy resin coatings, and offers a reliable and practical technical approach for the long-term corrosion prevention of metal equipment structural components.

## 2 Experimental

### 2.1 Materials

The coatings prepared in this study were primarily composed of metallic powders (Fe, Co, Ni, Cr, and Ti), novolac epoxy resin, curing agent, and dispersant. The o-cresol novolac epoxy resin (NPCN-704) used in the experiments was purchased from Nan Ya Plastics Corporation, Taiwan, China. The Fe, Co, Ni, Cr, and Ti powders were supplied by Lebo Metal Material Technology Co., Ltd with a purity of 99.99%. Specifically, the Co powder had a particle size of 3–5 µm, while Fe, Ni, Cr, and Ti powders were 300 mesh (approximately 48 µm). Dicyandiamide (analytical grade), anhydrous ethanol (analytical grade), and sodium chloride (analytical grade) were obtained from Macklin Biochemical Co., Ltd, Shanghai. The dispersant employed was a castor oil derivative (DK-20), procured from Guangdong Zhongke Hongtai Co., Ltd Q235 low-carbon steel was purchased from the local market. All chemical reagents were used directly without further purification.

### 2.2 Preparation of nano-sized high-entropy alloy Fe-Co-Ni-Cr-Ti

High-energy ball milling was adopted to prepare nano-sized high-entropy alloy powder fillers. The mass of mixed raw powders used in each milling jar was 7 g. Pure Fe, Co, Ni, Cr and Ti powders with an equimolar ratio of 1 : 1:1 : 1:1 were placed into an XQM-20 planetary ball mill (Changsha Tianchuang Powder Technology Co., Ltd, Changsha, China),where the mass ratio of milling balls to raw powder was fixed at 10 : 1. In addition, the diameters of large, medium and small milling balls are 12 mm, 8 mm and 5 mm, respectively, and their mass proportion was strictly controlled at a ratio of 1 : 2 : 10. Subsequently, an appropriate amount of ethanol was added, and nitrogen gas was introduced to provide an inert atmosphere. Nitrogen was adopted as the protective gas due to its reliable performance and long-term practical application in our laboratory, which can effectively prevent the oxidation of the powder during ball milling. The planetary ball mill was configured to run in a bidirectional alternating rotation mode, adopting a working cycle of 6 minutes combined with a 2 minutes pause interval. This intermittent operation mode was designed to prevent oxidation of the metal powders caused by excessive temperature rise. The total milling duration was 80 h.

Subsequent to milling, the alloyed powders were subjected to drying in a vacuum oven at 30 °C for 12 h, thereby producing nano-scale Fe-Co-Ni-Cr-Ti high-entropy alloy powder fillers. After drying, the powder was manually ground in an agate mortar to eliminate any agglomerates, and then sieved through a 100-mesh sieve to obtain a uniform particle size distribution, thereby producing nano-scale Fe-Co-Ni-Cr-Ti high-entropy alloy powder fillers.

### 2.3 Preparation of HEA/EPN powder coating

The HEA/EPN powder coating was fabricated *via* mechanical blending, in which the nano-sized Fe-Co-Ni-Cr-Ti HEA powder fillers were uniformly dispersed into the novolac epoxy resin matrix together with curing agent and dispersant. The total mass of the powder coating was 20 g. The phenolic epoxy resin and dicyandiamide curing agent with a mass ratio of 20 : 2 were placed in a beaker, 3 wt% of the dispersant was added, and 200 ml of deionized water was added. Mechanical stirring was performed at 25 °C using a JJ-1B constant-speed electric stirrer (60 W, manufactured by Xinyang Instrument Factory, Jintan District) at a rotation speed of 450 rpm (rpm). The mixture was stirred continuously for 2 h to achieve sufficient premixing. Then, different contents of Fe-Co-Ni-Cr-Ti alloy powders were added as fillers and mechanical stirring was continued for 10 hours. The contents of Fe-Co-Ni-Cr-Ti alloy were 1 wt%, 2 wt%, 3 wt%, and 4 wt% of the total mass of the powder coating, respectively, which were 0.2 g, 0.4 g, 0.6 g, and 0.8 g. After the mechanical blending was completed, the filter was used for filtration to remove the water, and the obtained precipitate was washed twice with water. Then, it was placed in a 25 °C forced air drying oven for drying for 12 hours. The powder after drying was ground until it could pass through a 100-mesh sieve and was ready for use. In addition, a pure phenolic epoxy resin coating without adding alloy was prepared as a control. Except for the filler content being 0 wt%, all the preparation processes were the same.

### 2.4 Preparation of HEA/EPN coatings

Q235 low-carbon steel served as the substrate, with specimens sized 7 cm × 8 cm. The substrate surface was ground stepwise using sandpapers with grit sizes ranging from 400 to 2000 mesh until it became smooth and free of oxide film. Subsequently, the ground samples were soaked in anhydrous ethanol and ultrasonically cleaned for 15 min to eliminate surface grease. After ultrasonic cleaning, the samples were taken out and placed in a blast drying oven, followed by drying at 60 °C for 30 min for further use.

**Fig. 1:**
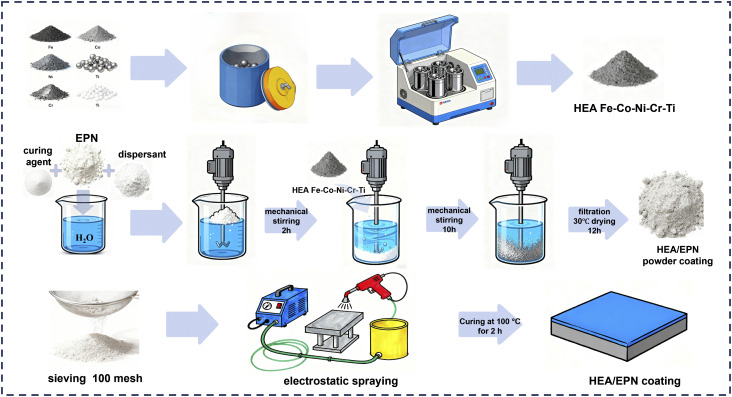
Schematic diagram of HEA/EPN coating preparation process.

Different contents of HEA/EPN powder coatings were applied onto the pretreated Q235 steel substrates *via* a commercial electrostatic spraying system (Model GH-JKPTJ-02 ZW, Jiangsu Guanghe Instrument Co., Ltd, China). The spraying and curing parameters were selected based on our previous work^35^ and further preliminary optimization experiments in this study. The process parameters of electrostatic spraying were configured as follows: an applied voltage of 60 kV was adopted with a spraying distance of 30 cm. After spraying, the coated samples were placed into a vacuum drying oven and cured at 160 °C for 2 h under atmospheric pressure, thus forming the HEA/EPN composite coatings. Fig. 1 schematically illustrates the preparation process of Fe-Co-Ni-Cr-Ti epoxy resin coatings on Q235 low-carbon steel substrates.

### 2.5 Characterization of HEA powders and HEA/EPN coatings

The phase compositions of the as-prepared HEA powders and HEA/EPN coatings were analyzed by X-ray diffraction (XRD, D8 ADVANCE) with Cu Kα radiation. The XRD tests were implemented with a scanning rate of 5° min^−1^ across a 2*θ* angle range of 10–90°.

The coating thickness was measured directly with a digital micrometer (Delixi Electric, measuring range: 0–25 mm, accuracy: ± 0.001 mm). For each coated sample, 8 distinct positions (including both central and edge areas) were selected for measurement to eliminate local deviations, and the arithmetic mean value was calculated as the final thickness.

The micro-morphologies and elemental distributions of the HEA powders and HEA/EPN coatings were characterized by a scanning electron microscope (SEM, Regulus 8220, Japan) equipped with an energy-dispersive X-ray spectroscopy (EDS) detector. For further analysis of the phase composition, crystal structure, and micro-morphology of the HEA powders, transmission electron microscopy (TEM, Tecnai G2F20, America) characterization was performed out at an accelerating voltage of 200 kV.

### 2.6 Electrochemical tests

To evaluate the passivation, barrier properties, and corrosion resistance of HEA/EPN coatings with different HEA contents in a 3.5 wt% NaCl solution, relevant measurements were conducted using an electrochemical workstation (Koster, CS35H0). The corrosive medium selected was 3.5 wt% sodium chloride solution, and a three-electrode system was adopted for the electrochemical tests: a platinum sheet was used as the counter electrode, a saturated calomel electrode (SCE) served as the reference electrode, and the prepared coatings served as the working electrode. The exposed surface area of each sample was strictly fixed at 1 cm^2^ by a self-made mold.

Electrochemical characterizations in this study consisted of open circuit potential (OCP) and electrochemical impedance spectroscopy (EIS) measurements, with the specific test procedures and parameters described as follows:

Electrochemical Impedance Spectroscopy (EIS) measurements were performed on samples immersed in a 3.5 wt% sodium chloride solution for durations ranging from 0 days (unimmersed) to 60 days. Prior to EIS measurements, each sample with different immersion times was subjected to an OCP test for 1800 s to achieve electrochemical system stabilization. Subsequently, EIS tests were performed out at the attained OCP with a 10 mV sinusoidal potential perturbation, over a frequency range of 10^5^ to 10^−2^ Hz. All electrochemical tests were replicated three times under identical conditions to ensure the validity and repeatability of the experimental data.

## 3 Results and discussion

### 3.1 Characterization of HEA Fe-Co-Ni-Cr-Ti

**Fig. 2:**
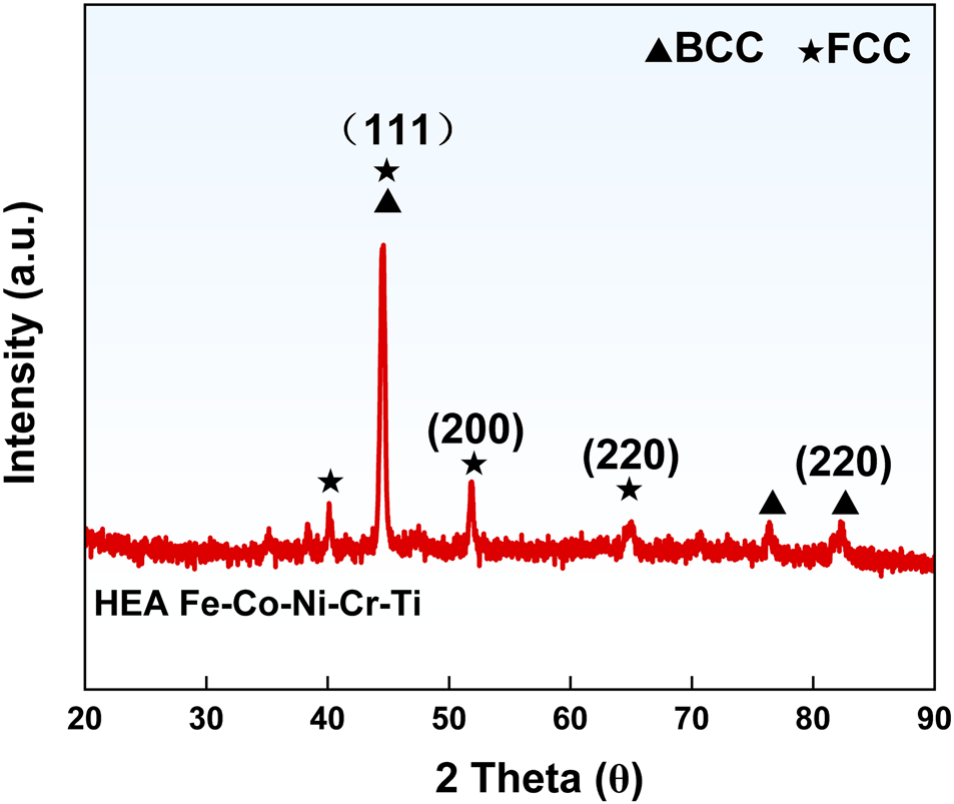
X-ray diffraction patterns of the HEA Fe-Co-Ni-Cr-Ti alloy powder.

Fig. 2 displays the XRD patterns of the Fe-Co-Ni-Cr-Ti HEA powder and HEA/EPN coating powder. The alloy has a dual-phase crystal structure (BCC + FCC), where Ti element promotes BCC phase formation. Peak intensity analysis shows that the FCC phase (111), (200) peaks are much stronger than the BCC phase (220) peak, confirming the FCC phase as the primary phase and BCC as the secondary phase in the HEA under the synergistic effect of Fe, Co, Ni, Cr, and Ti. The sharp diffraction peaks with narrow FWHM in the XRD patterns indicate no obvious amorphous characteristics or lattice distortion. The lattices of the FCC and BCC phases are regular, indicating good crystallinity of the alloy and good solid solution bonding of the five elements.

The nanoscale characteristics of the Fe-Co-Ni-Cr-Ti powder were confirmed using the Scherrer equation, which is expressed as follows:1
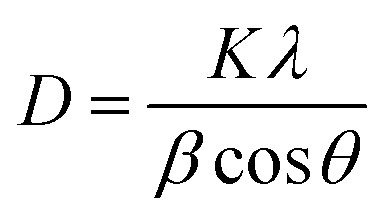
where *D* denotes the crystallite size, *K* represents the shape factor (∼0.9), *λ* is the X-ray wavelength (1.5406 Å for Cu Ka), *β* refers to the full width at half maximum (FWHM) in radians, and *θ* is the Bragg angle.^36^

After calculation, the crystallite size of the Fe-Co-Ni-Cr-Ti powder was determined to be 28.9 nm, confirming that the powder falls within the nanoscale range.

The lattice parameters of the FCC and BCC phases were calculated using Bragg's law (eqn (2)) and the relationship between interplanar spacing and lattice parameter (eqn (3)):

2 2*d*_*hkl*_ sin *θ* = *λ*

3
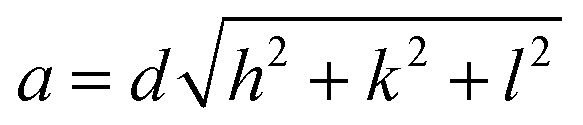
where *d*_*hkl*_ is the interplanar spacing, *θ* is the Bragg angle, *λ* is the X-ray wavelength (1.5406 Å for Cu Kα), and *a* is the lattice parameter. For the FCC phase, the (111) and (200) peaks were used, yielding an average lattice parameter of *a*_FCC_ ≈ 3.59 Å. For the BCC phase, the (220) peak was used, resulting in a lattice parameter of *a*_BCC_ ≈ 2.88 Å. These values are consistent with those reported for similar Fe-Co-Ni-Cr-Ti high-entropy alloys, confirming the formation of a stable dual-phase solid solution.

**Fig. 3:**
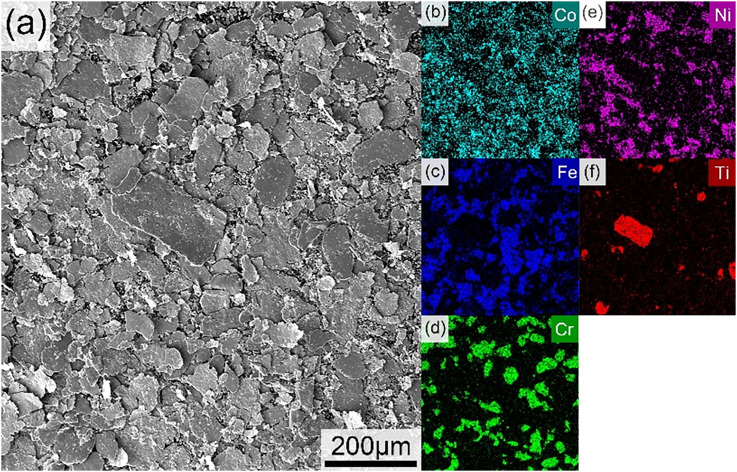
SEM images of (a) HEA Fe-Co-Ni-Cr-Ti; (b–f) EDS elemental mapping of Co, Fe, Cr, Ni, and Ti, respectively.

**Fig. 4:**
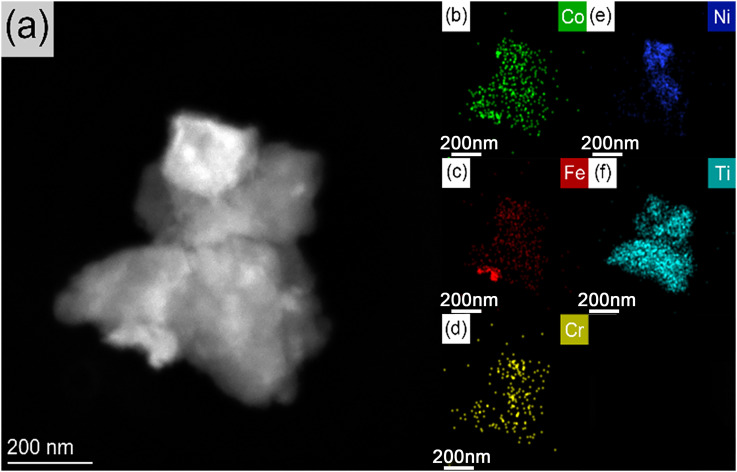
TEM images of (a) HEA Fe-Co-Ni-Cr-Ti; (b–f) EDS elemental mapping of Co, Fe, Cr, Ni, and Ti, respectively.

The surface microstructure and elemental composition of the Fe-Co-Ni-Cr-Ti high-entropy alloy were characterized by SEM and EDS, with the corresponding results presented in Fig. 3. The surface of the alloy powder fabricated *via* high-energy ball milling exhibits distinct sheet-like and layered structures, which are typical features of irregularly aggregated nanoscale particles, representing a typical ball milling “crushing-cold welding” process.^37,38^ These layered structures are closely arranged, with small interlayer gaps, overlapping to form tortuous corrosion paths, significantly extending the penetration distance of the corrosive medium. Meanwhile, the tiny interlayer gaps minimize the direct contact between the substrate and the corrosive medium, which plays a vital role in blocking the infiltration of the corrosive medium. The EDS results show that only five elements, Fe, Co, Ni, Cr, and Ti, were detected in the powder, with no obvious oxygen enrichment peaks or other heterogeneous impurity signals observed, which preliminarily indicates that no severe oxidation or obvious impurity introduction occurred during the preparation process. Among these elements, Fe, Co, and Ni elements exhibit macroscopic uniform distribution, and form a continuous matrix phase, whereas the Cr and Ti elements are present in the form of localized nanoscale enrichment, which appears as microscale agglomerates in the low-magnification SEM images (Fig. 3), as directly confirmed by the high-resolution TEM elemental mapping in Fig. 4. The Cr element exists in an island-like form, and the Ti element is a relatively continuous enrichment phase, satisfying “multi-component cooperative solid solution”. The absence of obvious oxide signals and impurities in this HEA can avoid forming preferential corrosion initiation sites and introducing additional chemical heterogeneous phases. This fundamentally reduces the formation of micro-cellular corrosion within the coating.

To further observe the fine microstructure of the Fe-Co-Ni-Cr-Ti alloy, transmission electron microscopy (TEM) characterizations, including bright-field morphology and EDS elemental mapping, were performed, with the results presented in Fig. 4. It is evident that the powder consists of aggregated nanoscale domains. The interfaces between adjacent nanoscale domains can be distinguished by contrast differences in the bright-field TEM image, which correspond to variations in local elemental distribution as shown in the EDS maps.

This nanoscale characteristic is the core requirement for the filler in the composite epoxy resin coating. On one hand, the nanoscale nature enables it to fill the voids within the coating and form a dense barrier protective layer; on the other hand, due to its larger specific surface area, the nanoscale filler can achieve better contact with the resin matrix, bond more tightly, enhance interfacial adhesion, and reduce corrosion channels.

The TEM-EDS elemental mapping further confirms that the powder consists solely of Fe, Co, Ni, Cr, and Ti, with no obvious oxygen enrichment peaks or other impurity signals, consistent with the SEM-EDS results. From the spatial distribution characteristics observed in the elemental maps and TEM bright-field image, Co and Ni exhibit a relatively uniform distribution across the entire nanoscale domains, with their distribution regions basically coinciding with the area of the nanoscale domains in the bright-field image, suggesting that they are the main components forming the continuous matrix of the alloy. Fe is distributed in localized concentrated regions within the nanoscale domains, with the size of these concentrated regions roughly in the nanoscale range, and the Fe signal intensity in these regions is significantly higher than that in the surrounding areas, indicating the presence of iron-rich nanoscale concentrated regions. Cr is distributed as discrete nanoscale concentrated features, which is related to its segregation tendency and limited diffusion during the ball milling process. Ti exists as continuous nanoscale enriched phases, partially overlapping with the concentrated regions of Cr and Fe, which may be a Ti-based composite phase.

This nanoscale alloy shows no obvious oxidation or impurity signals, as indicated by EDS characterization. On one hand, it ensures strong interfacial bonding with the epoxy resin matrix, forming a stable, tight interface and effectively mitigating interfacial corrosion. On the other hand, the absence of detectable impurity signals avoids the introduction of electrochemical heterogeneous phases, preventing “anode–cathode” microbattery formation and reducing internal electrochemical corrosion. The structural features—relatively uniform distribution of Co and Ni as the main matrix components, coupled with nanoscale dispersed distribution of Cr and Ti—provide a robust foundation for the alloy's excellent corrosion resistance. The multi-component alloy matrix, with its chemical inertness enhanced by the “high-entropy effect,” can form a continuous and dense passivation film on the surface, effectively blocking corrosive species such as Cl^−^, O_2_, and H_2_O.^21,39^ The nanoscale Cr features help repair passivation film defects, while the Ti-based composite phase acts as a physical barrier to extend the corrosion path, and the nanoscale dimensions fill resin matrix pores. Thus, the Fe-Co-Ni-Cr-Ti HEA acts as a functional filler, enhancing the coating's service life and corrosion resistance through synergistic effects of chemical inertness, physical barrier enhancement, and improved interfacial adhesion.

### 3.2 Morphological characterization of the HEA and coating

**Fig. 5:**
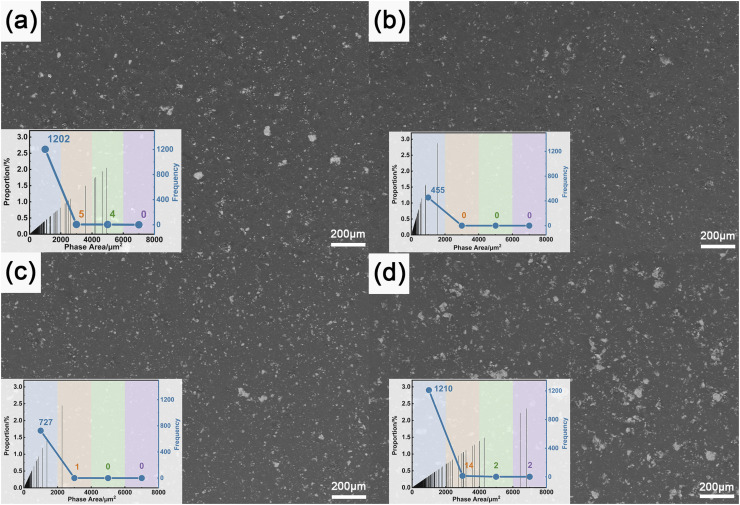
SEM images and corresponding agglomeration size distribution statistics of epoxy coatings with different HEA contents (white regions represent agglomerated phases): (a) 1 wt% HEA; (b) 2 wt% HEA; (c) 3 wt% HEA; (d) 4 wt% HEA.

Fig. 5 presents the SEM micrographs of HEA/EPN coatings with 1–4 wt% HEA content and pure epoxy resin coatings, integrated with the quantitative statistical results of agglomerates derived from these SEM images.

**Fig. 6:**
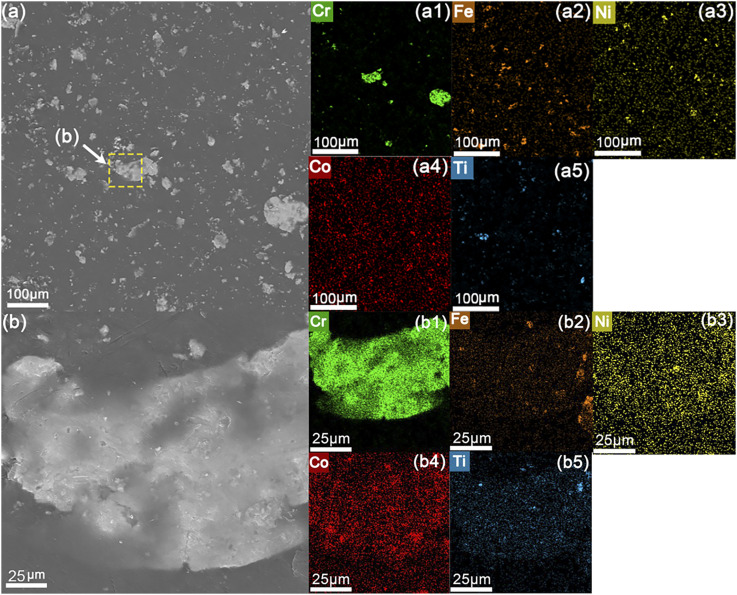
(a) EDS analysis of the epoxy coating with 4 wt% HEA content; (b) magnified view of the selected area in (a) and corresponding EDS results.

Fig. 6(a) is the full-view SEM-EDS image of the 4 wt% HEA-added coating (Fig. 5(d)), and Fig. 6(b) shows the locally magnified SEM-EDS characterization results of the agglomerated particles within the coating layer. From Fig. 5, it can be seen that all the coating surfaces present a uniform flat morphology, and there are no cracks or defects on the surface, indicating that the HEA filler has good compatibility with the phenolic epoxy resin matrix, and the addition of the coating does not cause the overall damage to the coating.

Based on the analysis of Fig. 5 and 6, it can be observed that all coating compositions exhibit a certain degree of particle agglomeration on their surfaces. According to EDS results, the matrix region of the coating appears in gray contrast, corresponding to the mixed phase of HEA fillers and the epoxy resin matrix. This is attributed to the fact that the epoxy resin consists mainly of low-atomic-number elements such as C, H, and O, which yield weaker secondary electron signals under SEM imaging, thereby presenting a darker gray contrast.

In contrast, the locally magnified view in Fig. 6(b) clearly reveals numerous bright white regions of varying sizes dispersed within the matrix. EDS analysis confirms that these bright areas consist primarily of high-entropy alloy elements, including Fe, Co, Ni, Cr, and Ti. These elements possess significantly higher atomic numbers compared to the matrix elements (C, H, O) and generate stronger secondary electron emission under electron beam excitation, resulting in distinct bright-white contrast in the images.

Moreover, the elemental composition of these bright regions matches well with the designed alloy formulation, indicating that these areas correspond to local physical agglomerates of HEA particles formed due to insufficient dispersion during their incorporation into the epoxy resin matrix. Although the HEA filler appears uniformly distributed in the epoxy matrix at the macroscopic scale, agglomeration and clustering resulting from interparticle interactions can still be observed at the micro-nanoscale. In summary, the bright white particles observed in Fig. 5 can be attributed to agglomerated regions of the nanoscale filler, highlighting the presence of localized particle aggregation within the composite coatings.

Fig. 5 reveals that the microstructural morphology and extent of particle agglomeration in the coatings vary with the filler content. Notably, the degree of particle agglomeration is one of the key factors restricting the microstructural uniformity and actual service performance of the coatings. Therefore, ImageJ software was employed to perform binarization processing on the scanning electron microscopy (SEM) images of the coatings. To ensure the reliability and repeatability of the statistical results, at least 5 non-overlapping visual fields were randomly selected for each coating sample, and the quantitative analysis was repeated three times independently. The error bars in the statistical results represent the standard deviation of the repeated measurements. Meanwhile, quantitative analyses were carried out for the area distribution, occurrence proportion, and frequency of agglomerated particles in the coating matrix, so as to elucidate the regulatory mechanism of HEA content on the agglomeration behavior of the coatings. The relevant statistical results have been overlaid in Fig. 5. In this figure, the white regions correspond to particle agglomerates, and the agglomerate area is divided along the horizontal axis into four ranges: <2000 µm^2^, 2000–4000 µm^2^, 4000–6000 µm^2^, and > 6000 µm^2^. The vertical axis represents the frequency of occurrence of agglomerates within each corresponding size range, and the black bars represent the proportion of agglomerates in each area interval.

When the HEA filler content is 1 wt%, the particles are sparsely dispersed as submicron-scale single entities, displaying a prominent “small-size enrichment” feature. Within the <2000 µm^2^ agglomeration area regime, the statistical frequency of agglomerated particles peaks at 1202 counts—corresponding to the predominant size fraction of the agglomerates. Only a marginal population of agglomerates is distributed in the 2000–4000 µm^2^ range (with a frequency of 5 counts), and no agglomerates are detected in the larger phase area intervals (4000–8000 µm^2^). These observations demonstrate that, at low HEA loadings, agglomeration within the coating is dominated by small-sized, high-density particle clusters, with no evidence of agglomerate coarsening behavior. The poor dispersion at 1 wt% can be attributed to the “concentration effect”: the low particle density leads to non-uniform spatial distribution, where local interparticle distances fall below the critical dispersion threshold, promoting the formation of dense small agglomerates despite the low overall content. These fine, dense small agglomerates create numerous nano-scale defects in the coating, which act as initial penetration channels for corrosive media (*e.g.*, Cl^−^), reducing the physical barrier effect of the coating to a certain extent.

As the HEA content increases further to 2 wt% and 3 wt%, the agglomeration distribution characteristics of the coating undergo a distinct transition: the frequency of small-sized agglomerates in the <2000 µm^2^ regime decreases to 455 and 727 counts, respectively. Only 1 count of agglomerates is detected in the 2000–4000 µm^2^ range for both compositions, and no agglomerates are observed in larger area intervals. These results indicate that 2 wt% and 3 wt% HEA fillers effectively suppress agglomerate formation without inducing large-sized agglomerates, with the 2 wt% formulation exhibiting the optimal dispersion performance. This improvement is driven by the “interfacial interaction effect”: an increased number of HEA particles forms stronger interfacial linkages (*e.g.*, hydrogen bonds, van der Waals forces) with active functional groups on the epoxy matrix, leading to full encapsulation of particles and enhanced steric hindrance, which effectively inhibits small-particle aggregation. The minimal agglomeration at 2 wt% minimizes the number of interfacial defects, forming a continuous and compact physical barrier that blocks the penetration of corrosive media; meanwhile, the uniformly dispersed HEA particles can form a continuous passive film on the coating surface, realizing the synergistic enhancement of physical barrier and chemical passivation protection, thus maximizing the corrosion resistance.

When the filler content is raised to 4 wt%, the frequency in the <2000 µm^2^ regime rebounds to 1210 counts, with 14 counts in the 2000–4000 µm^2^ range and 4 counts in the 4000–8000 µm^2^ interval. Both the number of agglomerated particles and their phase area dimensions are substantially higher than those of the other three formulations. This “decrease-rebound” trend suggests that there exists a critical threshold for HEA loading: beyond this threshold, the probability of small agglomerate formation increases again, accompanied by agglomerate coarsening and the emergence of a small number of large-sized agglomerates. This is because the filler content exceeds the dispersion capacity of the epoxy matrix, leaving some HEA particles unencapsulated and increasing their local collision probability, leading to both a rebound in small agglomerate frequency and the formation of large agglomerates *via* secondary aggregation. The large-sized agglomerates at 4 wt% form discontinuous “defect channels” in the coating matrix; corrosive media can rapidly penetrate the coating through these channels to reach the metal substrate, and the galvanic effect between the HEA agglomerates and the epoxy matrix further accelerates the local corrosion of the substrate, resulting in the poorest corrosion resistance among all samples.

SEM-EDS characterization combined with quantitative statistical results reveals that the agglomeration degree of the filler in the coating follows the order: 2 wt% < 3 wt% < 1 wt% < 4 wt%. This dispersion-dominated microstructure evolution is directly consistent with the electrochemical corrosion results: the minimal agglomeration at 2 wt% minimizes interfacial defects, forming an effective physical barrier against corrosive media and thus yielding the best corrosion resistance. Conversely, the severe agglomeration at 4 wt% creates abundant defect channels for corrosive species penetration, resulting in the poorest corrosion performance.

Notably, even at 4 wt% loading, no interface delamination induced by severe agglomeration is observed in the coating, indicating strong interfacial adhesion between the HEA filler and the phenol-formaldehyde epoxy matrix. This phenomenon lays a structural foundation for the stability of the coating's subsequent performance, such as its mechanical behavior and corrosion resistance.

### 3.3 Coating thickness measurement

Coating thickness is a key factors determinant of the barrier property, mechanical stability, and corrosion resistance durability in organic coatings. Variations in coating thickness can directly affect the electrochemical corrosion performance and the reliability of experimental results. A zonal measurement method was adopted to determine the thickness of coatings with different HEA contents. To ensure measurement accuracy, eight evenly distributed measuring points were selected on the surface of each coating sample (4 points in the central area and 4 points in the edge area), with the edge points being at least 5 mm away from the sample edge to avoid edge effects. Each point was measured twice, and the average value of the two measurements was taken as the effective thickness of that point. Finally, the arithmetic mean of the 7 points was calculated as the final thickness of the sample.

**Fig. 7:**
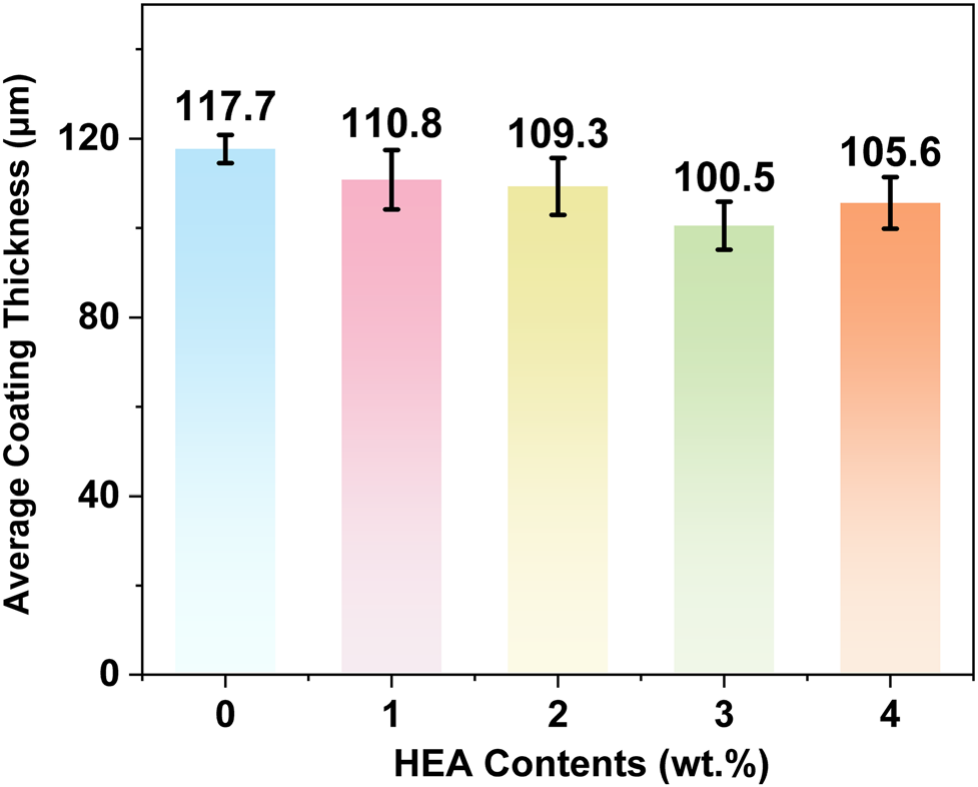
Thickness test results of HEA/EPN coatings with different HEA contents.

Fig. 7 presents the thickness test results of HEA/EPN coatings with varying HEA contents. The average thicknesses of the coatings with HEA contents ranging from 0 wt% to 4 wt% are 117.7 µm, 110.9 µm, 109.3 µm, 100.5 µm and 105.6 µm, respectively. All these average thickness values fall within the narrow range of 100.5–117.75 µm, which is well within the effective dry film thickness range (50–200 µm) for organic anti-corrosion coatings in engineering applications, as specified in the industrial standard.^40^ Notably, the thickness difference between distinct regions of each prepared coating sample is less than 5 µm, with no significant regional thickness deviation observed. This finding demonstrates that the as-prepared coatings possess excellent internal thickness uniformity.

**Fig. 8:**
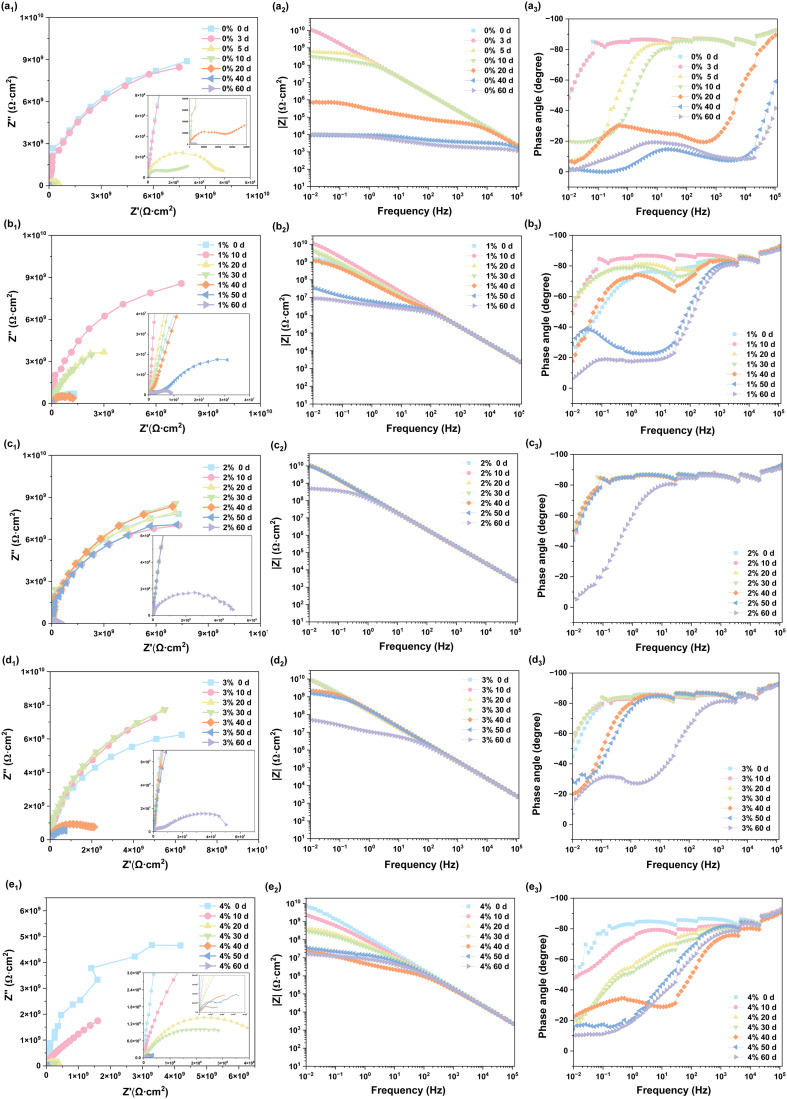
EIS plots of coatings with different immersion times: (a_1−_a_3_) neat epoxy resin; (b1–b3) 1 wt% HEA/EPN coatings; (c1–c3) 2 wt% HEA/EPN coatings; (d1–d3) 3 wt% HEA/EPN coatings; (e_1_–e_3_) 4 wt% HEA/EPN coatings.

The above test results further confirm that the electrostatic spraying process adopted in this study exhibits outstanding process stability and controllability. Even with the incorporation of different contents of nano-sized HEA reinforcing phases into the novolac epoxy resin matrix, a stable deposition rate and uniform spreading behavior were maintained throughout the spraying process. No local thickness mutation occurred as a result of uneven dispersion of the reinforcing phase powder or fluctuations in spraying parameters, and the thickness uniformity of the coatings was not adversely affected. This result eliminates the interference of coating thickness on the protective performance, thereby ensuring the reliability of subsequent electrochemical tests and immersion corrosion experiments (Fig. 8).

### 3.4 Corrosion resistance test of coatings: electrochemical impedance spectroscopy (EIS) measurements

Electrochemical tests were conducted on HEA/EPN coatings with 1 wt%, 2 wt%, 3 wt%, and 4 wt% fillers, as well as pure epoxy resin coatings, to study the influence of different filler contents on the corrosion resistance of the coatings. A simplified simulated seawater environment was constructed using a 3.5wt% NaCl monosalt system, and the experimental samples were subjected to 60 days long-term immersion. Electrochemical impedance spectroscopy (EIS) measurements were performed, with impedance spectra fitted to equivalent circuits. Electrochemical impedance spectroscopy (EIS) measurements were performed, and the obtained impedance spectra were fitted to appropriate equivalent circuits using ZView software, in accordance with the EIS interpretation methodology reported in.^41,42^

**Fig. 9:**
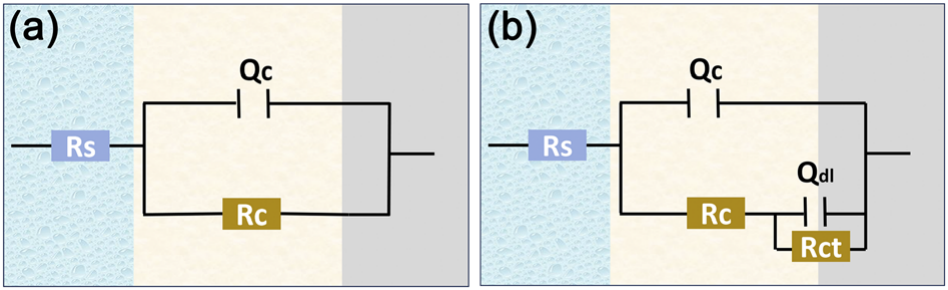
Equivalent circuit models for EIS fitting of HEA/EPN coatings: (a) single-time-constant model (intact coating stage); (b) two-time-constant model (medium penetration stage).

**Fig. 10:**
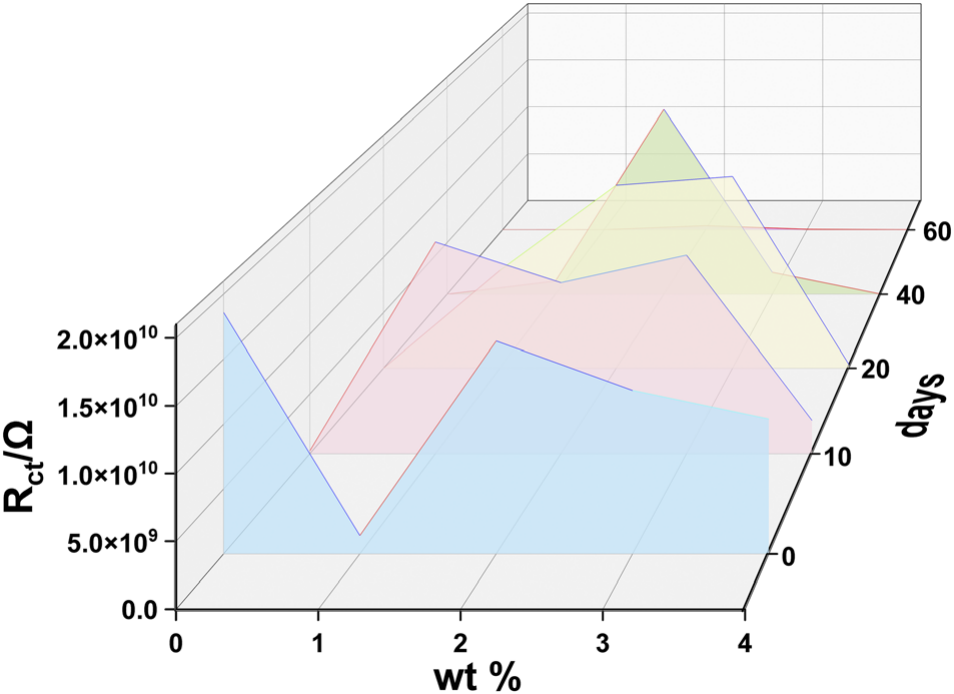
Comparison of *R*_c_ values of five coatings at different immersion times.

For coatings with an intact physical shielding effect (0–60 days for 2 wt% and 0–50 days 3 wt% HEA/EPN, early immersion stages for other coatings), a single-time-constant equivalent circuit *R*_s_−(CPE_1_−*R*_c_) was adopted, where *R*_s_ is the solution resistance, CPE_1_ the coating constant phase element, and *R*_c_ the coating resistance. For coatings where corrosive media had penetrated to the substrate/coating interface (*e.g.*, 20 days for pure EPN, 50 days for 1 wt% and 4 wt% HEA/EPN), a double-time-constant model *R*_s_−(CPE_1_−*R*_c_)–(CPE_2_−*R*_ct_) was used, with CPE_2_ representing the double-layer capacitance and *R*_ct_ the charge transfer resistance. The corresponding equivalent circuit fitting plots are shown in Fig. 9, and the fitting validity was verified by the consistency between the simulated and experimental curves.^41^Fig. 8 shows the Nyquist, Bode modulus, and phase angle plots of all coatings after 0–60 days of immersion, while EIS fitting parameters (including *R*_s_, CPE_1_, *n*_1_, *R*_c_) and calculated relative porosity are summarized in Tables 1–5. Fig. 10 compares the *R*_c_ values of the five coatings at different immersion durations, which is a key indicator for evaluating coating barrier performance.^43^

**Table 1:** EIS fitting parameters and relative porosity of the neat epoxy (EPN) coating

Sample	Immersion time (d)	*R*_s_/Ω cm^2^	CPE_1_		*R*_1_/Ω cm^2^	CPE_2_		*R*_2_/Ω cm^2^	Relative porosity (%)	*χ* ^2^/10^−3^
			(*Q*_1_)/10^−5^ Ω^−1^ cm^−2^ s^−*n*^	*n*_1_		(*Q*_2_)/10^−5^ Ω^−1^cm^−2^s^−*n*^	*n*_2_			
EPN	0 days	240.1	8.96 × 10^−5^	0.97	1.86 × 10^10^	—	—	—	1%	2.78
	3 days	207.6	8.65 × 10^−5^	0.96	1.74 × 10^10^	—	—	—	1.07%	1.98
	5 days	196.7	9.03 × 10^−5^	0.96	5.42 × 10^8^	—	—	—	34.32%	0.83
	10 days	361.2	1.05 × 10^−4^	0.95	2.37 × 10^8^	—	—	—	78.48%	3.13
	20 days	698.1	2.83 × 10^−4^	0.88	5.74 × 10^5^	8.54	0.42	2.57 × 10^6^	324.04%	4.39
	40 days	216.3	1.79 × 10^−4^	0.92	3640	1.31	0.53	24 261	5109%	2.66
	60 days	312.5	5.14 × 10^−4^	0.84	1961	3.54	0.44	12 858	9484%	0.95

**Table 2:** EIS fitting parameters and relative porosity of the 1 wt% HEA/EPN coating

Sample	Immersion time (d)	*R*_s_/Ω cm^2^	CPE_1_		*R*_1_/Ω cm^2^	CPE_2_		*R*_2_/Ω cm^2^	Relative porosity (%)	*χ* ^2^/10^−3^
			(*Q*_1_)/10^−5^ Ω^−1^ cm^−2^ s^−*n*^	*n*_1_		(*Q*_2_)/10^−5^ Ω^−1^ cm^−2^s^−*n*^	*n*_2_			
1 wt% HEA/EPN	0 days	1724	2.04 × 10^−4^	0.88	1.41 × 10^9^	—	—	—	13.19%	3.10
	10 days	1072	9.25 × 10^−5^	0.96	1.77 × 10^10^	—	—	—	1.05%	1.63
	20 days	2416	1.84 × 10^−4^	0.89	8.81 × 10^9^	—	—	—	2.11%	3.78
	30 days	1906	2.22 × 10^−4^	0.87	8.97 × 10^9^	—	—	—	2.07%	8.08
	40 days	2985	3.26 × 10^−4^	0.82	1.288 × 10^9^	—	—	—	14.44%	4.78
	50 days	1747	1.53 × 10^−4^	0.91	6.01 × 10^6^	0.016	0.73	1.53 × 10^7^	309.48%	5.04
	60 days	285.1	1.24 × 10^−4^	0.94	2.08 × 10^6^	0.013	0.31	5.37 × 10^6^	894.23%	6.03

**Table 3:** EIS fitting parameters and relative porosity of the 2 wt% HEA/EPN coating

Sample	Immersion time (d)	*R*_s_/Ω cm^2^	CPE_1_		*R*_1_/Ω cm^2^	CPE_2_		*R*_2_/Ω cm^2^	Relative porosity (%)	*χ* ^2^/10^−3^
			(*Q*_1_)/10^−5^ Ω^−1^ cm^−2^ s^−*n*^	*n*_1_		(*Q*_2_)/10^−5^ Ω^−1^ cm^−2^ s^−*n*^	*n*_2_			
2 wt% HEA/EPN	0 days	1909	9.42 × 10^−5^	0.96	1.64 × 10^10^	—	—	—	1.13%	1.30
	10 days	1025	9.41 × 10^−5^	0.96	1.43 × 10^10^	—	—	—	1.30%	1.01
	20 days	1344	9.54 × 10^−5^	0.96	1.65 × 10^10^	—	—	—	1.13%	1.35
	30 days	1140	9.53 × 10^−5^	0.96	1.82 × 10^10^	—	—	—	1.02%	1.87
	40 days	1233	9.78 × 10^−5^	0.95	1.79 × 10^10^	—	—	—	1.04%	1.31
	50 days	1007	9.55 × 10^−5^	0.96	1.71 × 10^10^	—	—	—	1.09%	1.09
	60 days	758	1.51 × 10^−4^	0.87	4.27 × 10^8^	—	—	—	45.36%	1.25

**Table 4:** EIS fitting parameters and relative porosity of the 3 wt% HEA/EPN coating

Sample	Immersion time (d)	*R*_s_/Ω cm^2^	CPE_1_		*R*_1_/Ω cm^2^	CPE_2_		*R*_2_/Ω cm^2^	Relative porosity (%)	*χ* ^2^/10^−3^
			(*Q*_1_)/10^−5^ Ω^−1^ cm^−2^ s^−*n*^	*n*_1_		(*Q*_2_)/10^−5^ Ω^−1^ cm^−2^ s^−*n*^	*n*_2_			
3 wt% HEA/EPN	0 days	243.4	1.05 × 10^−4^	0.95	1.26 × 10^10^	—	—	—	1.48%	1.11
	10 days	234.7	1.22 × 10^−4^	0.93	1.66 × 10^10^	—	—	—	1.12%	1.44
	20 days	342.6	1.14 × 10^−4^	0.94	1.73 × 10^10^	—	—	—	1.08%	1.39
	30 days	261.9	1.13 × 10^−4^	0.95	1.75 × 10^10^	—	—	—	1.06%	1.37
	40 days	207.8	1.00 × 10^−4^	0.93	2.13 × 10^9^	—	—	—	8.73%	1.84
	50 days	440.2	1.01 × 10^−4^	0.94	1.12 × 10^9^	—	—	—	16.61%	2.04
	60 days	628.2	1.88 × 10^−4^	0.90	7.99 × 10^6^	0.049	0.59	6.02 × 10^7^	232.79%	4.76

**Table 5:** EIS fitting parameters and relative porosity of the 4 wt% HEA/EPN coating

Sample	Immersion time (d)	*R*_s_/Ω cm^2^	CPE_1_		*R*_1_/Ω cm^2^	CPE_2_		*R*_2_/Ω cm^2^	Relative porosity (%)	*χ* ^2^/10^−3^
			(*Q*_1_)/10^−5^ Ω^−1^ cm^−2^ s^−n^	*n*_1_		(*Q*_2_)/10^−5^ Ω^−1^ cm^−2^ s^−*n*^	*n*_2_			
4 wt% HEA/EPN	0 days	440.7	1.11 × 10^−4^	0.91	1.04 × 10^10^	—	—		1.79%	4.26
	10 days	1107	2.32 × 10^−4^	0.74	2.79 × 10^9^	—	—	—	6.67%	6.42
	20 days	1077	1.07 × 10^−4^	0.93	3.44 × 10^8^	—	—	—	54.07%	1.25
	30 days	1297	4.21 × 10^−4^	0.82	2.41 × 10^8^	—	—	—	77.18%	4.94
	40 days	572.5	2.68 × 10^−4^	0.64	1.54 × 10^7^	—	—	—	1207.79%	7.77
	50 days	610.7	2.06 × 10^−4^	0.90	1.12 × 10^7^	2.51	0.21	2.26 × 10^7^	1660%	3.51
	60 days	104.4	4.69 × 10^−4^	0.80	7.73 × 10^6^	1.48	0.58	1.16 × 10^7^	2406%	4.76

The protective performance of HEA/EPN coatings is highly dependent on both HEA loading and immersion time, and the phase angle frequency characteristics directly reflect the dynamic transformation of the coating's anti-corrosion mechanism, consistent with the EIS interpretation principles reported in. At the initial stage (0 days), all coatings exhibited a single continuous phase angle plateau over a wide frequency range, corresponding to a single time constant feature. This behavior is attributed to the dense organic composite structure formed by the epoxy matrix and HEA fillers, which exerts an effective physical shielding effect and blocks the corrosive media from contacting the Q235 carbon steel substrate.^44^ With the extension of immersion time, the phase angle plateau split into high- and low-frequency dual regions, a typical characteristic indicating the penetration of corrosive media to the coating/substrate interface; consequently, the protective mechanism transformed into a synergy of “HEA passivation + physical shielding”. The passivation effect is primarily derived from the multi-component nature of HEA fillers, which tend to form a compact passive film composed of Cr_2_O_3_ and TiO_2_ on the coating surface (etailed characterization and verification of this passive film, including its chemical composition and microstructure, will be presented in our subsequent work).

Among all coatings, the 2 wt% HEA/EPN coating maintained the widest phase angle plateau throughout the 60 days immersion, with a low-frequency phase angle still above 65° at 60 days, indicating the optimal corrosion resistance and interface stability. In contrast, the phase angle plateau of the pure EPN coating shrank rapidly after 30 days, with a low-frequency phase angle below 45° at 60 days, suggesting the complete loss of physical shielding effect.

The capacitive arc radius of the Nyquist plot, the low-frequency impedance modulus (|*Z*| at 0.01 Hz) in the Bode plot, coating constant phase element (CPE_1_) and relative porosity are synergistic indicators for characterizing coating corrosion resistance and structural stability.^41,42^ The capacitive arc radius is positively correlated with the coating impedance value, and the |*Z*| at 0.01 Hz directly reflects the long-term barrier capability of the coating. The CPE_1_ value is associated with the dielectric properties of the coating: an increase in CPE_1_ indicates the infiltration of electrolyte into the coating, leading to an increase in the coating dielectric constant, while the decrease in the phase index *n*_1_ reflects the aggravation of coating surface inhomogeneity and defect formation. Relative porosity, calculated based on *R*_c_ with pure EPN at 0 days as the reference, quantitatively characterizes the defect degree of the coating: a higher relative porosity means more corrosive medium penetration pathways and poorer barrier performance.

The pure EPN coating had an initial *R*_c_ of 1.86 × 10^10^ Ω cm^2^ and a relative porosity of 1.00% (Table 1). With increasing immersion time, its *R*_c_ decreased rapidly, while CPE_1_ (coating constant phase element) and relative porosity increased significantly: the relative porosity rose to 34.32% at 5 days, indicating the onset of severe coating degradation, and *R*_c_ dropped to 5.57 × 10^6^ Ω cm^2^ at 20 days. At 60 days, the |*Z*| at 0.01 Hz was below 12 858 Ω cm^2^, and the relative porosity reached 9484.96%, demonstrating the complete failure of the pure EPN coating due to the absence of HEA reinforcement.

Notably, the 1 wt% HEA/EPN coating exhibited a unique “densification” behavior during the initial immersion stage (Table 2): its relative porosity decreased sharply from 13.19% at 0 days to 1.05% at 10 days, accompanied by a simultaneous decrease in CPE_1_ and an increase in the phase index *n*_1_ to 0.96. This phenomenon indicates that the low-content HEA fillers filled the micro-gaps in the epoxy matrix through physical filling and interfacial interactions, thereby enhancing coating compactness and mitigating electrolyte infiltration. However, after 40 days, the *R*_c_ of the 1 wt% HEA/EPN coating decreased significantly, and the relative porosity soared to 14.44%; at 60 days, *R*_c_ was only 5.37 × 10^6^ Ω cm^2^, and |*Z*| at 0.01 Hz was below 1 × 10^7^ Ω cm^2^. This performance degradation is attributed to initial defects induced by a small number of large HEA agglomerates, which provided pathways for corrosive medium penetration and resulted in inferior interfacial stability compared with the 2 wt% and 3 wt% HEA/EPN coatings.

The 2 wt% HEA/EPN coating exhibited the most outstanding long-term corrosion resistance (Table 3): its *R*_c_ remained at around 10^10^ Ω cm^2^ in the first 50 days, with the relative porosity stably below 1.13% and CPE_1_ almost unchanged (maintained at ∼9.5 × 10^−5^ Ω^−1^ cm^−2^ s^−*n*^), indicating a dense and stable coating structure with minimal electrolyte infiltration. Even at 60 days, its *R*_c_ still remained at 4.27 × 10^8^ Ω cm^2^, the relative porosity was only 43.56%, and |*Z*| at 0.01 Hz was 5 × 10^8^ Ω cm^2^ (a decrease of less than two orders of magnitude compared with 0 days). This is attributed to the uniform dispersion of nano-sized HEA fillers in the epoxy matrix, which not only fills the micro-voids to form a denser physical shielding layer but also forms a continuous passive film on the coating surface; the optimal synergy of these two effects endows the coating with excellent long-term barrier performance.

The 3 wt% HEA/EPN coating showed good corrosion resistance in the early immersion stage (Table 4): its *R*_c_ remained at 10^10^ Ω cm^2^ within 30 days, with a relative porosity below 1.48%, which is comparable to that of the 2 wt% HEA/EPN coating. After 40 days, its *R*_c_ decreased to 2.13 × 10^9^ Ω cm^2^ (relative porosity 8.73%), and the performance decayed faster than the 2 wt% coating, which is ascribed to the formation of small isolated HEA agglomerates. However, at 60 days, its *R*_c_ was still 6.02 × 10^7^ Ω cm^2^ (relative porosity 232.79%), which is much higher than those of the pure EPN, 1 wt% and 4 wt% HEA/EPN coatings, indicating that the isolated agglomerates did not form connected defects and still provided effective protection.

When the HEA loading was increased to 4 wt%, the 4 wt% HEA/EPN coating exhibited the poorest corrosion resistance (Table 5): although its initial *R*_c_ was 1.04 × 10^10^ Ω cm^2^ (relative porosity 1.79%), its *R*_c_ decreased sharply and CPE_1_ increased significantly after 10 days; at 20 days, the relative porosity soared to 54.07%, and the capacitive arc showed a “bimodal” feature, a typical indication of the early penetration of corrosive media. At 60 days, its *R*_c_ was only 1.16 × 10^6^ Ω cm^2^, and the relative porosity reached 2406.21%. This severe performance degradation is due to the large number and large size of HEA agglomerates caused by excessive loading, which destroyed the integrity of the epoxy matrix, formed abundant connected corrosion channels, and significantly impaired the physical shielding effect of the coating.

Overall, the improvement of the long-term corrosion resistance of epoxy coatings by Fe-Co-Ni-Cr-Ti HEA is not a simple linear relationship, but is highly dependent on the uniform dispersion of HEA fillers and the resulting coating defect degree (characterized by CPE_1_ and relative porosity).^41,42^ The 2 wt% HEA/EPN coating achieves the optimal long-term corrosion resistance through the synergistic mechanism of atomic-scale homogeneous dispersion, dense composite structure, and multi-component passivation protection, retaining a high *R*_c_ of 4.27 × 10^8^ Ω cm^2^ even after 60 days of immersion. The 3 wt% HEA/EPN coating has slightly inferior performance due to small isolated agglomerates, while the 1 wt% and 4 wt% HEA/EPN coatings exhibit poor long-term stability caused by HEA agglomeration. All HEA/EPN coatings show better corrosion resistance than the pure EPN coating, indicating that the introduction of nano-sized HEA fillers is an effective strategy to enhance the long-term barrier performance of epoxy coatings.

### 3.5 Corrosion resistance mechanism of HEA/EPN coating

**Fig. 11:**
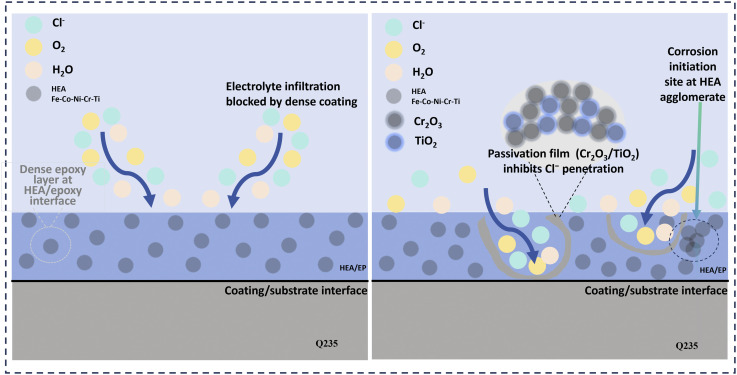
Corrosion resistance mechanism of HEA/EPN coatings.

From the above analysis, it can be concluded that the addition of high-entropy alloy Fe-Co-Ni-Cr-Ti nanoparticles significantly enhances the shielding effect and corrosion resistance stability of the epoxy resin coating through “structure optimization-element synergy-passivation protection”, and greatly extends the corrosion resistance and protection period of the coating. Fig. 11 presents the corrosion mechanism diagram of the HEA/EPN coating. Its protection process is a typical “two-stage synergistic protection”, namely the initial shielding protection stage and the later anode polarization protection stage,^45–47^ and the protective mechanisms at each stage are closely correlated with the coating microstructure and the passivation mechanism of HEA.

Initially, the uniformly distributed nano-sized high-entropy alloy fillers can efficiently fill the original pores and micro-defects of the epoxy resin coating, forming a continuous and dense structure. This dense coating structure can prevent corrosive media such as Cl^−^, O_2_, and H_2_O from penetrating the coating surface, reducing the penetration path and providing a physical shielding protective effect.

During prolonged continuous immersion, the coating surface corrosion penetration depth increases steadily with time. When corrosive medium penetration reaches the critical threshold, the coating barrier performance gradually fails. The corrosive medium will gradually penetrate into the interior of the coating and even completely penetrate to the coating/bottom interface. At this point, the protection mechanism enters the passivation synergistic protection stage. At this time, HEA Fe-Co-Ni-Cr-Ti plays a key role. On the one hand, the structural optimization of the shielding effect and the *in situ* formation of the passivation film form a dense protection. The Cr element, as the key component of passivation, first undergoes selective oxidation in the corrosive medium, and combines with Ti and Fe elements to form a Cr_2_O_3_-TiO_2_-Fe_3_O_4_ composite passivation film. This passivation film is dense and exhibits high chemical stability,^7,32,39^ which can effectively block further contact between the corrosive medium and the substrate, thus inhibiting continuous anodic dissolution. Among them, Ti element doping not only refines the grain size of the passivation film, but also can optimizes the microstructure characteristics and enhances the interfacial bonding strength between the passivation film and the substrate. This prevents the passivation film from cracking or detaching under stress and extends the duration of passivation protection. In addition, multiple elements can cooperatively regulate the corrosion kinetics process. The solid solution strengthening effects of Ni and Co refine the grains of the HEA. Although grain refinement increases the total grain boundary area, the uniform elemental distribution and rapid formation of a dense passivation film effectively inhibit corrosion propagation along grain boundaries. This improves the thermodynamic stability of the coating and reduces its overall electrochemical activity in 3.5 wt% NaCl solution. The synergy between Ni and Cr effectively promotes Cr enrichment in the passivation film, further enhancing its density and dissolution resistance. Co can boost the electronic conductivity of the passivation film and suppress the occurrence of cathode reduction reactions. In addition, the potential difference among the elements forms a uniformly distributed “micro-battery effect”. The easily passivated Cr and Ti preferentially dissolve and passivate, indirectly protecting Fe, Co, and Ni, and slowing the overall corrosion propagation rate.

It is worth noting that the dispersion state of HEA directly determines the effectiveness of the protective mechanism: the uniform dispersion of 2 wt% HEA enables a more continuous formation of the passivation film, resulting in the most uniform distribution of the battery, and the optimal synergy between the physical shielding effect and the passivation protection. Therefore, after 60 days, it still maintains the highest *R*_ct_ value; the slight isolated agglomeration of 3 wt% HEA does not damage the overall continuity of the passivation film and can also maintain a certain synergistic protective effect; while the local agglomeration and connected agglomeration of 1 wt% and 4 wt% lead to the local rupture of the passivation film or the inability to form continuously during long-term service, and the corrosive medium quickly penetrates through the gaps of the agglomeration, ultimately causing the failure of the coating protection.

In summary, the Fe-Co-Ni-Cr-Ti/EPN composite coating achieves long-term and high-efficiency corrosion protection *via* a two-stage synergistic mechanism of physical barrier-passivation protection: the structural optimization of epoxy resin constructs an initial protective barrier, while the multi-element synergy of HEA generates a stable passive film, thus forming a double-layer protective system. This system significantly enhances the overall corrosion resistance and long-term service stability of the coating, with 2 wt% HEA identified as the optimal addition level for this system.

## 4 Conclusion

In this study, Fe-Co-Ni-Cr-Ti high-entropy alloy (HEA) powders were prepared by high-energy ball milling. HEA/EPN coatings were fabricated on the surface of carbon steel through mechanical blending and electrostatic spraying. The effects of different HEA addition amounts (0 wt%, 1 wt%, 2 wt%, 3 wt%, 4 wt%) on the microstructure of the coatings and their corrosion resistance in 3.5 wt% NaCl were systematically investigated. The corrosion protection mechanism was clarified. The main conclusions are as follows:

(1) The dense sheet-like HEA powder with uniform elemental distribution acts as an effective long-term corrosion-resistant filler, prolonging the penetration path of corrosive media and suppressing localized corrosion.

(2) HEA enhances coating corrosion resistance *via* a synergistic “physical shielding – multi-element passivation” mechanism: nano-sized HEA fills epoxy voids to strengthen shielding, while *in situ* Cr_2_O_3_/TiO_2_ passivation film and optimized grain structure form a dual protective system.

(3) HEA agglomeration dominates long-term performance, with 2 wt% being the optimal loading. At this content, uniform dispersion yields the highest coating resistance (*R*_c_ = 4.27 × 10^8^ Ω cm^2^) after 60 days of immersion. All HEA-reinforced coatings outperform pure epoxy, with 3 wt% showing mild decline and 1 wt%/4 wt% exhibiting significant degradation due to agglomeration.

While this study clarifies the corrosion behavior of HEA/EPN coatings in static NaCl solution, future work will explore their performance under dynamic marine conditions (*e.g.*, flow, wave impact) and complex corrosive environments (*e.g.*, acidified seawater, SRB). Further investigation will also focus on the growth kinetics of Cr_2_O_3_-TiO_2_ passive films and interfacial interactions between HEA and epoxy resin.

## Author contributions

Xinyue Fan performed the experiments and wrote the initial manuscript. Yaqian Liu and Guojun Ji designed the research, supervised the work and revised the manuscript. Guojun Ji is the principal corresponding author. All authors have approved the final version of the manuscript.

## Conflicts of interest

The authors declared that they have no conicts of interest to this work.

## Data Availability

The data that support the findings of this study are available from the corresponding author upon reasonable request. These data include long-term corrosion resistance test results, surface morphology images of the coatings, and mechanical property characterization data of the high-entropy alloy reinforcements.
